# Epididymal anomalies in boys with undescended testis or hydrocele: Significance of testicular location

**DOI:** 10.1186/s12894-015-0099-1

**Published:** 2015-10-24

**Authors:** Sun-Ouck Kim, Seong Woong Na, Ho Song Yu, Dongdeuk Kwon

**Affiliations:** From the Department of Urology, Chonnam National University Medical School, Gwangju, South Korea

**Keywords:** Undescended testis, Epididymis, Hydrocele

## Abstract

**Background:**

Epididymal anomalies and patent processus vaginalis are frequently found in boys with cryptorchidism or hydrocele. We conducted this study to evaluate the association between epididymal anomalies and testicular location or patent processus vaginalis in boys with undescended testis or hydrocele.

**Methods:**

Children undergoing surgery with undescended testis (group A, 136 boys and 162 testes) or communicating hydrocele (group B, 93 boys and 96 testes) were included. Testicular locations and epididymal anomalies were investigated prospectively. An anomalous epididymis was defined as anomalies of epididymal fusion that consisted of loss of continuity between the testis, the epididymis, and the long looping epididymis. The epididymis was considered normal when a normal, firm attachment between the testis, the caput, and the cauda epididymis was present.

**Results:**

The mean ages of groups A and B were 24.6 ± 19.7 (range, 8–52 months) and 31.4 ± 20.6 months (range, 10–59 months). The incidence of epididymal anomalies was significantly higher in group A than that in group B (65.4 % vs. 13.5 %, *P* < 0.001). The incidence of epididymal anomalies in boys with undescended testis was significantly different according to testis location. Epididymal anomalies were observed in 100 %, 91.4 %, and 39.3 % of cases when the testis was located in the abdomen, inguinal canal, and distal to the external inguinal ring, respectively (*P* < 0.001).

**Conclusion:**

We conclude that epididymal anomalies were more frequent in boys with undescended testis than in boys with hydrocele, and that these anomalies were more frequent when undescended testis was at a higher level. These results suggest that testicular location is associated with epididymal anomalies rather than patent processus vaginalis.

## Background

Epididymal and vasal anomalies are associated with undescended testis. These anomalies occur in association with undescended testis at varying degrees of 32–79 % according to various epididymal anomaly diagnostic criteria [1–7]. These anomalies can be found either during orchiopexy or hydrocelectomy/hernia repair. The patency of processus vaginalis is also strongly related with epididymal anomalies [8].

The surgeon must be aware of associated epididymal anomalies when operating to correct undescended testes or hydrocele and not to dissect an elongated epididymis, a cranially located testis with a descended long or dissociated epididymis, or a cranially located testis [5]. Although epididymal anomalies in children with undescended testis or hydrocele are detected frequently during the surgery, only limited and sporadic reports are available on this subject.

Some reports have suggested that epididymal anomalies associated with more cranially located testis are more severe than those associated with more caudally located testis among boys with undescended testis [4]. However, no study has evaluated epididymal anomalies based on the location of undescended testis, which represents the exact incidence rate. We prospectively evaluated the relationship between epididymal anomalies in boys with undescended testis or hydrocele. The aim of this study was to clarify whether patent processus vaginalis or undescended testis is a more important factor in epididymal anomalies. We specially focused on the severity of epididymal anomalies according to testicular location in boys with undescended testis to better understand the relationship between epididymal morphology and testicular descent.

## Methods

### Patients and study design

We included children undergoing an inguinal exploration for undescended testis or a communicating hydrocele in the urologic department of our hospital between January 2011 and July 2013. A communicating hydrocele was defined by the size change history and a sac extending to the groin on ultrasound sonography. We performed a cross-sectional study to determine the incidence of epididymal anomalies in these children. Boys with any of the following were excluded from the study: associated disorders, such as hypospadias, exstrophy, imperforated anus, sex development disorders, ectopic testes, or vanishing testis. The spermatic cord was isolated after incising the cremasteric fascia, and the hernia sac was high-ligated when present. Orchiopexy was performed by placing the testis into the subdartos pouch. Hydrocelectomy was performed using a typical inguinal approach, and the testis was delivered to the groin so the epididymal anomaly could be examined. Written informed consent for data collection and participation in the study was obtained from a parent or guardian. This study received approval from the local ethics committee (CNUH institutional review board). The study procedures complied with the guidelines provided by the Declaration of Helsinki.

### Definition of epididymal anomalies

Testicular locations and epididymal anomalies were investigated carefully. According to morphological classification by Barthold and Redman [9], an anomalous epididymis was defined as an anomaly of epididymal fusion consisting of loss of continuity between the testis and the epididymis or long looping epididymis. The epididymis was considered normal when a normal firm attachment between the testis and the caput and cauda epididymis was present.

### Statistical analyses

SPSS ver. 17 for Windows software (SPSS Inc., Chicago, IL, USA) was used for statistical analyses. Data were analyzed with the chi-square test. *P*-values < 0.05 were deemed significant.

## Results

A total of 229 boys (258 testes) with undescended testis (group A: 136 boys and 162 testes) or with hydrocele (group B: 93 boys and 96 testes) were included in this study. The mean ages in groups A and B were 24.6 ± 19.7 (range, 8–52 months) and 31.4 ± 20.6 months (range, 10–59 months), respectively. In total, 110 patients (left: 52, right: 58) in group A had unilateral lesions and 26 patients had bilateral lesions. A total of 90 boys in group B had unilateral lesions (left: 55, right: 35) and three had bilateral lesions. The patient demographic data are shown in Table 1.

**Table 1:** Patient characteristics

	Group A	Group B
Mean age (months)	24.6 ± 19.7	31.4 ± 20.6
No. of boys (n)	136	93
No. of testis (n)	162	96
Bilaterality (n)		
Lt.	52	55
Rt.	58	35
Bilateral	26	3

No. = number, Lt = left, Rt = right, Group A: undescended testis, Group B: hydrocele

The incidence of epididymal anomalies was significantly higher in group A than that in B (65.4 % [106/162] vs. 13.5 % [13/96], *P* < 0.001) (Table 2). The incidence of epididymal anomalies in group A was 100 % (20/20) when the affected testis was found in the abdomen, 91.4 % (53/58) when it was found in the inguinal canal and 39.3 % (33/84) when it was found in the distal to external inguinal ring. This result indicates that higher testicular locations were associated with higher frequencies of epididymal anomalies (*P* < 0.001) (Table 3).

**Table 2:** Type of epididymis in cases of undescended testis and hydrocele

Type of epididymis	No. of boys (%)	
	Group A	Group B
Normal epididymis	56 (34.6)	83 (86.5)
Normal firm attachment	32 (19.8)	45 (46.9)
Widening of the mesentery	24 (14.8)	38 (39.6)
Abnormal epididymis^a^	106 (65.4)	13 (13.5)
Complete separation of the caput	27 (16.7)	0 (0)
Complete separation of the cauda	32 (19.8)	7 (7.3)
Complete separation of the caput and cauda	20 (12.3)	6 (6.3)
Long looping type	27 (16.7)	0 (0)

No. = number, Group A: undescended testis, Group B: hydrocele. ^a^Fisher’s exact test, *P* = 0.001

**Table 3:** Epididymal anomalies according to location of undescended testis

Location of testis	Epididymal anomalies No. of testis (%)	*P*-value
Intra-abdominal	20/20 (100)	
Inguinal canal	53/58 (91.4)	<0.001*
Distal to external inguinal ring	33/84 (39.3)	
Total	106/162 (65.4)	

*No* number; *Fisher’s exact test

## Discussion

We evaluated the associations between epididymal anomalies and testicular location or patent processus vaginalis in boys with undescended testis or hydrocele, specially focusing on severity of the epididymal anomalies according to testicular location in boys with undescended testis. The incidence of epididymal anomalies was significantly higher in boys with undescended testis (65.4 %) than in boys with hydrocele (13.5 %).

The estimated overall incidence of epididymal anomalies in boys with undescended testis and hydrocele was 46.1 %, suggesting that testicular location contribute more to epididymal anomalies than patent processus vaginalis. Epididymal anomalies were found more frequently in boys with a higher testicular location. All boys with undescended testis located in intra-abdominal area had epididymal anomalies, 91.4 % when the undescended testis was in the inguinal canal, and 39.3 % when it was distal to external inguinal ring.

Controversies exist regarding the role of the epididymis in testicular descent, as epididymal anomalies have been found in 36–79 % of boys with undescended testis. Most studies that have reported anatomical abnormalities of epididymis in boys with undescended testis have been sporadic and used different methods, and no recent data were found [1–7]. Mollaeian et al. reported that epididymal and vas anomalies occur with an overall frequency of 36 % in boys with undescended testis [7]. Gill et al. reported that epididymal anomalies were detected in 43 % of boys with undescended and vanishing testis [4]. In the present study 65.4 % of boys with undescended testis had epididymal anomalies. The reason for the discrepancy may be due to different evaluation tools used in the studies. We used the most recently reported and most widely used epididymal anomaly classification [9].

Factors that have been suggested as important for normal testicular descent include normal gubernaculum, epididymis, and intra-abdominal pressure, and innervation by the genitofemoral nerve of the gubernaculums [10, 11]. However, some boys with normally descended testis have epididymal and vassal anomalies [12]. Elder evaluated the incidence of epididymal anomalies based on patency of the processus vaginalis in patients with hernia, hydrocele, and undescended testis and suggested that most epididymal abnormalities probably do not contribute to the testicular descent process [6]. That author insisted that an abnormal epididymis with undescended testis maybe an associated finding and not a cause, as he reported that 64 % of cases showed an epididymal anomaly when the processus vaginalis was completely patent, whereas 11 % were abnormal when the processus vaginalis was incompletely patent [6]. We found a rather low incidence of boys with patent processus vaginalis compared to that in Elder’s study. We found that 13.3 % of boys with a communicating hydrocele and completely patent processus vaginalis and normally descended testis had epididymal anomalies. This discrepancy may be due to different inclusion criteria for the studies. We compared the incidence of epididymal anomalies between boys with normal descended testis with patent processus vaginalis/hydrocele and boys with undescended testis.

Knowledge of normal testicular anatomy is necessary to understand epididymal anomalies. The normal epididymis curves along the testis but the epididymal body may be slightly detached from the testis body. The epididymal head remains in close proximity with the cranial pole of the testis, while the cauda epididymis disappears within the gubernaculum [13]. The testis and caput epididymis arise from the genital ridge, whereas the body of the epididymis and vassal structure arise from the mesonephric tubules and Wolffian duct. Then, union of the rete testis and mesonephric tubules begins at 12 weeks of gestation. During involution of the mesonephric duct, the testis receives blood from the internal spermatic artery, and the vessel structures derive blood supply from the internal iliac artery. Ductal or epididymal atresia or segmental anomalies may occur die to a vascular accident related to this difference in vascular supply [14].

Epididymal anomalies are associated with maldescent of the testis and may account for impaired future fertility [3, 4]. A clinically important point to consider is possible infertility due to epididymal or vassal malformation of the sperm transporting mechanism. Infertility problems in patients with undescended testis may partly result from obstruction associated with an abnormal epididymis [3, 4]. Koff and Scaletscky reported that an elongated epididymis may present problems for sperm maturation and transportation, and may be associated with severe impairment in future fertility, despite early surgical correction of undescended testis [5].

In the present study, epididymal anomalies were detected in all boys when the affected testis was found in the intra-abdominal area, whereas 91.4 % showed an abnormal condition when the testis was in the inguinal canal and 39.3 % when it was found distal to the external inguinal ring. More severe epididymal anomalies and vassal anomalies are encountered in boys with testis located in the intra-abdominal or high inguinal position in a small case series [1, 4]. Our results suggest that higher testicular locations were associated with more frequent epididymal anomalies, as shown previously. It appears that future fertility issue should be considered when epididymal anomalies are detected at orchiopexy, particularly when the affected testis is located in a high cranial position.

## Conclusions

We conclude that epididymal anomalies were more frequent in boys with undescended testis than in boys with hydrocele, and that these anomalies were more frequent when the undescended testis was at a higher level. This result suggests that testicular location, rather than patent processus vaginalis, is associated with epididymal anomalies.
